# MiR-384 inhibits human colorectal cancer metastasis by targeting KRAS and CDC42

**DOI:** 10.18632/oncotarget.12704

**Published:** 2016-10-17

**Authors:** Yong-Xia Wang, Yan-Ru Chen, Shan-Shan Liu, Ya-Ping Ye, Hong-Li Jiao, Shu-Yang Wang, Zhi-Yuan Xiao, Wen-Ting Wei, Jun-Feng Qiu, Li Liang, Wen-Ting Liao, Yan-Qing Ding

**Affiliations:** ^1^ Department of Pathology, Nanfang Hospital, Southern Medical University, Guangzhou, Guangdong, China; ^2^ Department of Pathology, School of Basic Medical Sciences, Southern Medical University, Guangzhou, Guangdong, China; ^3^ Guangdong Province Key Laboratory of Molecular Tumor Pathology, Guangzhou, Guangdong, China; ^4^ Department of Pathology, School of Basic Medical Sciences, Xinxiang Medical University, Xinxiang, Henan, China

**Keywords:** colorectal cancer, MiR-384, metastasis, KRAS, CDC42

## Abstract

Colorectal cancer (CRC) is the third most common cancer worldwide. Metastatic progression is a primary factor contributing to lethality of CRC patients. However, the molecular mechanisms forming early local invasion and distant metastatic colonies are still unclear and the present therapeutic approaches for CRC are unsatisfactory. Therefore, novel therapies targeting metastatic invasion that could prevent tumor spreading and recurrence are urgently needed. Our study showed that the decrease of miR-384 was found in 83.0% (83/100) CRC patients. And low-leveled expression of miR-384 was closely correlated with the invasive depth, lymph node and distant metastasis of CRC. Overexpression of miR-384 could inhibit the invasive and migrating abilities of CRC cells *in vitro* and the metastatic potential *in vivo*. Luciferase assays showed that miR-384 repressed the expression of Kirsten Ras (KRAS) and Cell division cycle 42 (CDC42) by directly targeting their 3’-untranslated regions. There is functional and mechanistic relationship between miRNA-384 and KRAS, CDC42 in the invasion and metastasis of CRC. And our findings suggest that miR-384could be a potent therapeutic target for CRC. Restoration of miR-384 expression might provide novel therapeutic approach to the reduction of CRC metastasis.

## INTRODUCTION

Colorectal cancer (CRC) is the third most common cancer and one of the major causes of death globally [1]. CRC accounts for about 10% of all cancer cases with over a million new cases diagnosed worldwide annually [2]. Death from CRC is mainly due to metastatic progression, and the liver is the main organ of metastatic colonization in over 70% of patients [3, 4]. 50-60% of CRC patients can develop distant metastases and the 5-year survival rate will decrease to 5% in patients with distant metastases [5, 6]. Up to now, efforts to prevent metastasis and increase cure rates after surgery have focused on combined chemotherapy administration. Unfortunately, such therapy is unsatisfactory in reducing metastatic relapse. The high prevalence and lack of effective therapeutic targets of this disease demand a greater understanding of its biological progression [7].

In the past few years, the molecular alterations in CRC have been studied extensively. For example, microsatellite instability, CpG island methylator phenotype, PIK3CA, RAS and BRAF mutations have become routine clinical practice in determining CRC treatment [8, 9]. The molecular alterations such as RAS or BRAF mutations in CRC patients affect the clinical therapeutic effects [10, 11]. However, a more detailed pathway deregulated in CRC should be described for improving clinical management. Ras protein regulates many signaling pathways by affecting a cascade of downstream proteins, such as PI3K-AKT, PI3K-Rho-CDC42, Raf-MEK-Erk and Ral-CDC42, et al [12, 13]. KRAS and CDC42 are important members of Ras signaling pathways and play an important role in the invasion and metastasis of tumor [14].

In recent years, microRNAs (miRNAs), which are small noncoding RNAs, have been verified to be silenced or over-expressed and to act to suppress or promote metastatic progression in many cancer types [15–17]. MiRNAs negatively regulate gene expression by directly binding to the 3'-untranslated region (3'UTR) of their target mRNAs. For example, miR-1246 promotes cancer stemness including self-renewal, drug resistance, tumorigencity and metastasis by activation of Wnt/β-catenin pathway via suppressing the expression of AXIN2 and GSK3β [18]. MiR-361-5p and miR-30a are down-regulated in CRC and their over-expression inhibits the progression of CRC by directly binding to the 3'UTR of their target genes [19, 20]. The role of miRNAs as molecular probes for the identification of metastasis regulators as well as therapeutic targets of CRC has much better application prospects and has become the research focus. MiRNAs, with the tendency of accumulating in hepatic tissue, promise more efficacy in suppressing liver metastatic colonization [21]. Therefore, identification of therapeutic miRNAs would be of greatly clinical value.

Studies have implicated that miR-384 (miR-384-3p) might serve as tumor suppressor in some human tumors [22–24]. But little is known about the roles of miR-384 in the clinical pathological correlations and biological functions in the process of CRC carcinogenesis and development. Our research will delineate the role of miR-384 in the metastasis of CRC and explore a new therapeutic target for CRC metastasis.

## RESULTS

### Down-regulation of miR-384 is closely related with the aggressive characteristics of CRC

**Figure 1:**
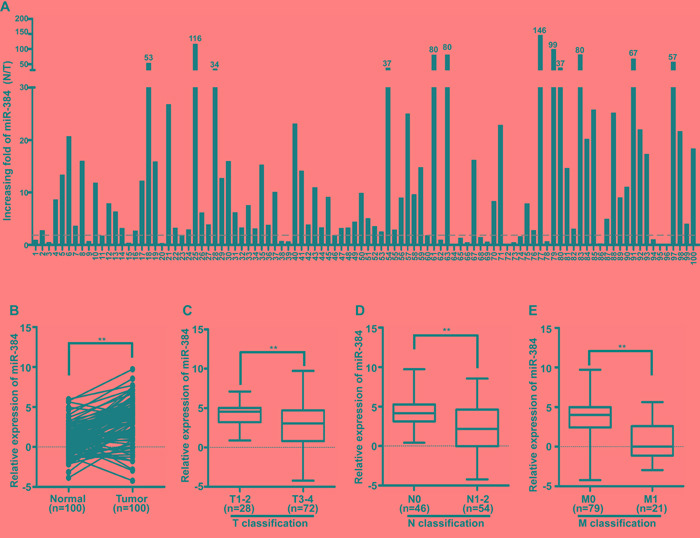
MiR-384 is down-regulated in CRC patients **A.** Expression of miR-384 in 100 cases of fresh human CRC tissues and their matched adjacent normal tissues by real-time PCR analyses; miR-384 expression was normalized to U6 and expressed relative to the matched adjacent normal tissues (2^-ΔΔCт^). **B.** Mean expression of miR-384 in 100 cases of fresh human CRC tissues and their matched adjacent normal tissues by real-time PCR (2^-ΔCт^, n=100, ^**^p<0.01). **C.** Mean expression of miR-384 by real-time PCR according to the T classification (2^-ΔCт^, n=100, ^*^p<0.05). **D.** Mean expression of miR-384 by real-time PCR according to the N classification (2^-ΔCт^, n=100, ^**^p<0.01). **E.** Mean expression of miR-384 by real-time PCR according to M classification (2^-ΔCт^, n=100, ^**^p<0.01). Boundaries of boxes represent bounding of the boxes and stand for the lower and upper quartile. Lines within the boxes and whiskers represent median and extremum (maximum and minimum).

The expression of miR-384 was detected in 100 cases of fresh primary CRC biopsies and their paired adjacent normal tissues by real-time PCR. We found that miR-384 was down-regulated in 83.0% (83/100) of CRC tissue samples (T) compared to their matched adjacent normal tissues (N). A twofold difference (N/T>twofold) accounted for 75.0% (75/100) (Figure 1A). Student's t-test showed that the expression level of miR-384 in CRC tissues samples was significantly lower than that in adjacent normal tissues (Figure 1B).

In addition, we investigated the clinicopathological significance of miR-384 expression in CRC patients. The median relative expression level of miR-384 in 100 cases of CRC samples was taken as the cut-off point to separate tumors with low expression of miR-384 from those with high expression of miR-384. Mann-Whitney U tests and Spearman correlation analysis suggested that expression of miR-384 was significantly associated with the differentiation degree, T classification (invasive depth), N classification (lymph node metastasis) and M classification (distant metastasis) of CRC (Table 1 and Supplementary Table S1; Figure 1C-1E). But there were no significant correlation between the expression level of miR-384 and the patients' age and gender (Table 1).

**Table 1:** Clinicopathologic characteristics of miR-384 expression in CRC patients

Clinicopathological variables	miR-384 Expression		*P* Value
	Low	High	
**Age (years)**			
<60	23	26	0.688
≥60	26	25	
**Gender**			
Male	29	24	0.227
Female	20	27	
**Differentiation**			
Well	34	3	0.000
Moderate	15	36	
Poor	0	12	
**T classification**			
T_1-2_	20	8	0.005
T_3-4_	29	43	
**N classification**			
Yes	19	35	0.003
No	30	16	
**M classification**			
Yes	4	17	0.002
No	45	34	

### Over-expression of miR-384 inhibits the invasive and metastatic abilities of CRC cells *in vitro* and *in vivo*

**Figure 2:**
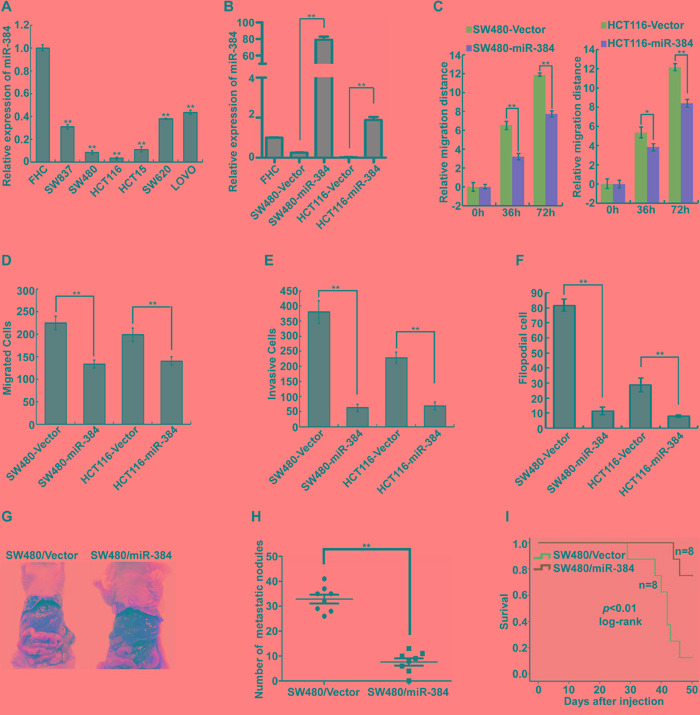
Over-expression of miR-384 inhibits the invasive and metastatic abilities of CRC cells *in vitro* and *in vivo* **A.** Expression of miR-384 in six CRC cell lines and one normal colorectal mucosa cell line FHC were detected by real-time PCR. **B.** Over-expression of miR-384 in SW480 and HCT116 CRC cells verified by real-time PCR. **C.** Wound-healing assay. Histograms represent the average migrated distances at the indicated times. Error bars represent mean±s.d. from three independent experiments. **D-E.** The migratory and invasive properties of SW480/Vector, SW480/miR-384 and HCT116/Vector, HCT116/miR-384 cells were analyzed using Boyden chambers or Matrigel-coated Boyden chambers. Error bars represent mean±s.d. from three independent experiments. **F.** Three-dimensional morphologies assay. Histograms represent the average number of filopodia formed by each cell sphere from three independent experiments. Error bars represent mean±s.d.. **G.** Representative images of gross specimens of liver metastatic lesions formed in mice injected intrasplenically with SW480/Vector and SW480/miR-384. **H.** The number of liver visible metastatic nodules was shown (^**^p<0.01). (I) Overall survival of mice bearing liver metastases of SW480/Vector and SW480/miR-384 (log-rank test, n=8, ^**^ *p*<0.01).

The expression of miR-384 was examined in six CRC cell lines and one normal colorectal mucosa cell line FHC by real-time PCR. It was found that the expression of miR-384 was significantly lower in six CRC cell lines than in FHC (Figure 2A). And among the six CRC cell lines, the expression of miR-384 was relatively lower in SW480 and HCT116 than that in SW620 and LOVO (Figure 2A). To evaluate the possible functions of miR-384 in CRC progression, we transfected the CRC cell lines SW480 and HCT116 with hsa-miR-384 mimics and obtained the cells with overexpressed miR-384(Figure 2B). Then, we observed the effects of miR-384 in migration and invasion of CRC cells by Wound-healing assay, Transwell migration assay, Matrigel invasion assay and three-dimensional morphogenesis assay, and found that the over-expression of miR-384 inhibited the migration and invasion ability of CRC cells *in vitro* (Figure 2C-2F and Supplementary Figure S1A-S1D). To further verify that miR-384 could inhibit the metastasis of CRC cells *in vivo*, control vector and miR-384 over-expressed SW480 (SW480/Vector and SW480/miR-384) cells were injected intrasplenically. The results showed that the mice injected with SW480/miR-384 cells exhibited less visible metastatic nodules in the liver than SW480/Vector group (Figure 2G). Histological staining confirmed that the nodules in the liver were metastatic CRC (Supplementary Figure S1E). The number of liver visible metastatic nodules in SW480/miR-384 group was significantly less than that in SW480/Vector (Figure 2H). CRC cells with over-expressed miR-384 markedly extended the overall survival of the mice (Figure 2I).

### Inhibition of endogenous miR-384 promotes the invasive and metastatic abilities of CRC cells *in vitro* and *in vivo*

**Figure 3:**
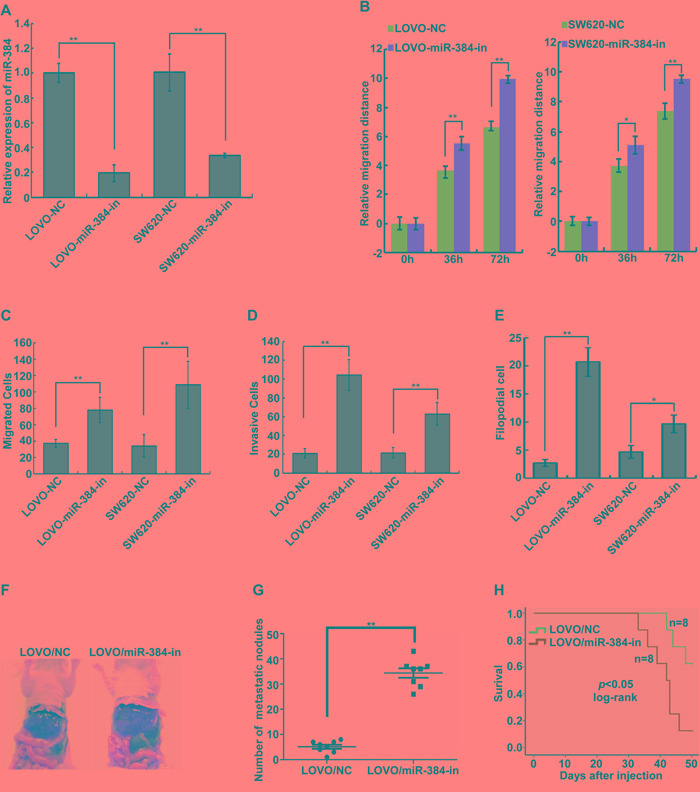
Inhibition of endogenous miR-384 promotes the invasive and metastatic abilities of CRC cells *in vitro* and *in vivo* **A.** Expression of miR-384 in LOVO and SW620 CRC cells transfected with inhibitor or their paired negative control lentiviral vector was detected by real-time PCR. **B.** Wound-healing assay. Histograms represent the average migrated distances at the indicated times. Error bars represent mean±s.d. from three independent experiments. **C-D.** The migratory and invasive properties of LOVO/NC, LOVO/miR-384-in and SW620/NC, SW620/miR-384-in cells were analyzed using Boyden chambers or Matrigel-coated Boyden chambers. Error bars represent mean±s.d. from three independent experiments. **E.** Three-dimensional morphologies assay. Histograms represent the average number of filopodia formed by each cell sphere from three independent experiments. Error bars represent mean±s.d.. **F.** Representative images of gross specimens a of liver metastatic lesions formed in mice injected intrasplenically with LOVO/NC and LOVO/miR-384-in. **G.** The number of liver visible metastatic nodules was shown(^**^p <0.01). **H.** Overall survival of mice bearing liver metastases of LOVO/NC and LOVO/miR-384-in (log-rank test, n=8, ^**^ *p*<0.01).

Endogenous expression of miR-384 was inhibited in LOVO and SW620 by transfecting miR-384 inhibitors (Figure 3A). We detected the migratory and invasive abilities of the above cells by Wound-healing assay, Transwell migration assay, Matrigel invasion assay and three-dimensional morphogenesis assay. The results showed that suppression of miR-384 significantly increased the migration and invasion abilities of LOVO and SW620 cells compared to their negative control (NC) transfected cells (Figure 3B-3E and Supplementary Figure S2A-S2D). To further observe the inhibition effects of miR-384 on the metastasis *in vivo*, stable low miR-384 expressing LOVO (LOVO/miR-384-in) cells and the control (LOVO/NC) cells were injected intrasplenically. Results showed that LOVO/miR-384-in group demonstrated more visible metastatic nodules in the liver than that in LOVO/NC group (Figure 3F-3G and Supplementary Figure S2E). And the overall survival of the mice was significantly shortened with the inhibition of miR-384(Figure 3H).

### MiR-384 decreases KRAS and CDC42 expression by directly binding to their 3’UTR in CRC cells

**Figure 4:**
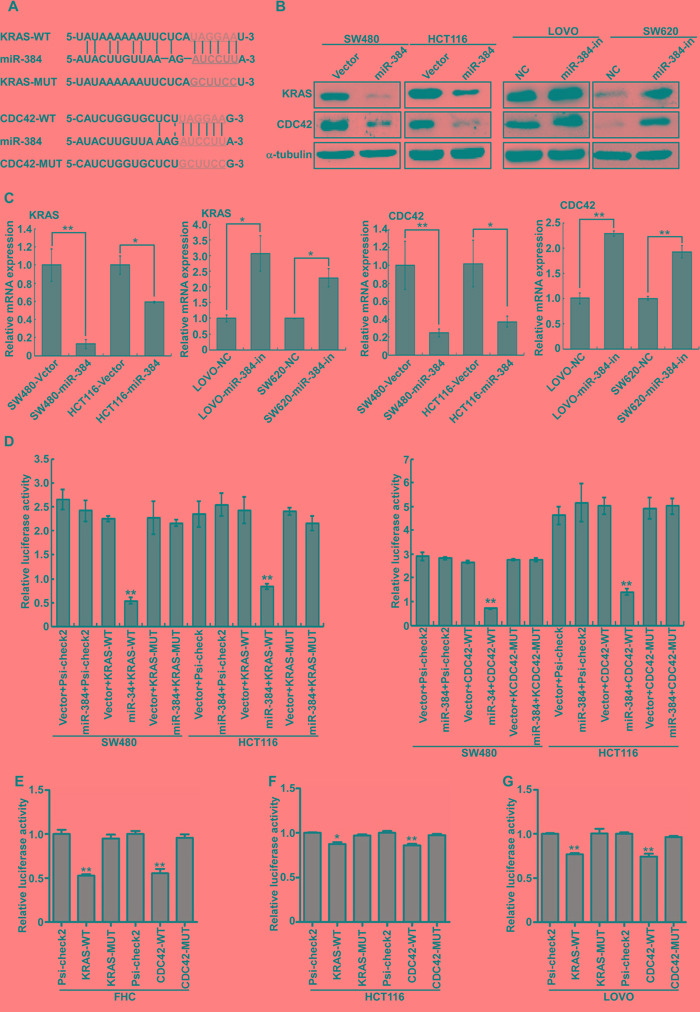
KRAS and CDC42 are direct targets of miR-384 **A.** Predicted miR-384 target sequences in the 3’UTRs of KRAS and CDC42, and their mutants containing altered nucleotides in the 3’UTRs. **B.** Western blot analysis of KRAS and CDC42 in the indicated cells. **C.** Real-time PCR analysis of KRAS and CDC42 mRNA expression. **D.** Luciferase assay analyses of the indicated cells transfected with the indicated reporters with miR-384 (Error bars represent mean ± SD from three independent experiments; ^**^p< 0.01). **E-G.** Luciferase assay analyses with the variable endogenous levels of mir-384 in FHC, HCT116 and LOVO cell lines (Error bars represent mean ± SD from three independent experiments; ^*^p< 0.05, ^**^p< 0.01).

To further explore the function and mechanism of miR-384, it is necessary to determine an mRNA target of miR-384 that might mediate the role of miR-384 in the invasion and metastasis of tumor. Three publicly available bioinformatic algorithms (TargetScan, Pictar, miRANDA) were used to analyze target genes of miR-384(Supplementary Figure S3A). The results indicated that KRAS and CDC42 were theoretical target genes of miR-384 (Figure 4A). Therefore, KRAS and CDC42 mRNA and protein expression in miR-384 over-expressed or miR-384-downregulated cells were investigated respectively. Real-time PCR and western blot analyses showed that the mRNA and protein levels of KRAS and CDC42 were significantly down-regulated in miR-384 over-expressed cells, whereas they were up-regulated after inhibition of miR-384 (Figure 4B-4C).

To confirm that KRAS and CDC42 were directly inhibited by miR-384, the dual-luciferase reporter system was performed. Firstly, we subcloned the KRAS and CDC42 3'UTR fragments containing miR-384 binding site and their mutant fragments into the psi-CHECK2 luciferase reporter vectors. It was found that the co-expression of miR-384 markedly inhibited the renilla luciferase reporter activity of the wild-type KRAS and CDC42 3'UTR, but did not change the activity of the mutant 3'UTR constructs and their scramble vectors (Figure 4D). Luciferase assay analyses with the variable endogenous levels of mir-384 in FHC, HCT116 and LOVO cell lines also demonstrated that miR-384 inhibited KRAS and CDC42 expression via binding to their 3'UTR (Figure 4E-4G).

### Repression of KRAS and CDC42 plays an important role in miR-384-inhibited invasion and metastasis of CRC cells

**Figure 5:**
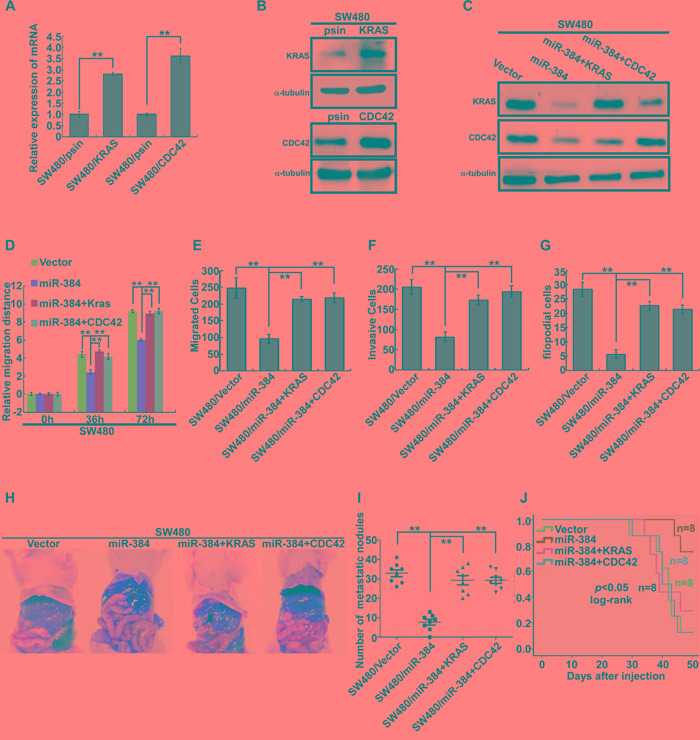
Repression of KRAS and CDC42 plays an important role in miR-384-inhibited invasion and metastasis of CRC cells **A-C.** KRAS and CDC42 over-expression in SW480 cells by real-time PCR analysis or Western blot. **D.** Wound-healing assay. Histograms represent the average migrated distances at the indicated times. Error bars represent mean±s.d. from three independent experiments. **E-F.** The migratory and invasive properties of SW480/Vector, SW480/miR-384 and SW480/miR-384+KRAS, SW480/miR-384+CDC42 cells were analyzed using Boyden chambers or Matrigel-coated Boyden chambers. Error bars represent mean±s.d. from three independent experiments. **G.** Three-dimensional morphologies assay. Histograms represent the average number of filopodia formed by each cell sphere from three independent experiments. Error bars represent mean±s.d.. **H.** Representative images of gross specimens of liver metastatic lesions formed in mice injected intrasplenically with SW480/Vector, SW480/miR-384, SW480/miR-384+KRAS and SW480/miR-384+CDC42. **I.** Number of visible metastatic nodules in the liver. ^**^p<0.01. **J.** Overall survival of mice bearing liver metastases (log-rank test, n=8, ^*^p <0.05).

**Figure 6:**
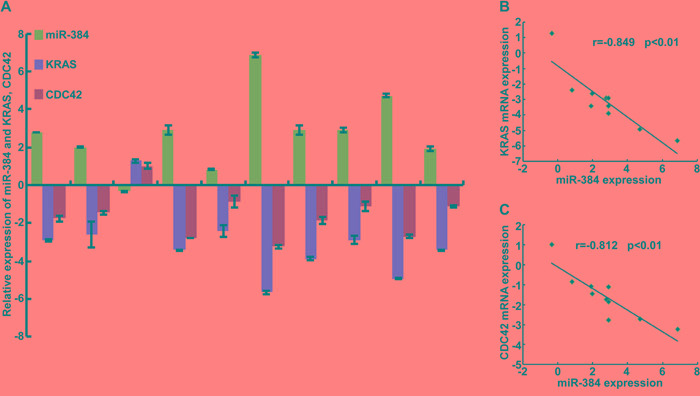
Correlation between the expression of miR-384 and KRAS or CDC42 in CRC tissues **A.** The expression of miR-384 and KRAS, CDC42 were detected by real-time PCR(ΔCт, n=10). **B.** Spearman correlation analyses of miR-384 expression and KRAS mRNA expression. **C.** Spearman correlation analyses of miR-384 expression and CDC42 mRNA expression.

To further confirm the role of miR-384 in the progression of CRC, we next restored the expression of KRAS and CDC42 in miR-384 over-expressed SW480 cells (Figure 5A-5C) by transfection of KRAS(G12V) and CDC42 ORF constructs without 3'UTRs, and observed their effects on invasion and migration. The results showed that the invasive and migrated abilities of miR-384 over-expressed SW480 cells increased with the restoration of KRAS and CDC42 (Figure 5D-5G and Supplementary Figure S3B-S3E). Moreover, with the restoration of KRAS (G12V) and CDC42, the metastatic abilities of miR-384 over-expressed SW480 *in vivo* also increased significantly (Figure 5H-5J and Supplementary Figure S3F). Then the correlation between the expression of miR-384 and KRAS or CDC42 was analyzed in 10 freshly collected CRC biopsies to further verify whether the above findings could be supported in human primary tumors tissues. The results showed that KRAS or CDC42 expression was negatively correlated with the expression of miR-384 (Figure 6A-6C). Meanwhile, we found that there was positive correlation between the expression KRAS and CDC42 (Supplementary Figure S3G-S3H).

## DISCUSSION

CRC is one of the major lethal causes of cancer patients with more than one million of newly diagnosed cases a year globally. Among these patients, more than 600,000 ones are in advanced stage with liver metastasis [25]. Tumor invasion and metastasis is a complex and multistep process that is regulated by many molecules, including miRNAs [26, 27]. Metastasis, especially liver metastasis, has been identified as the major poor prognostic factor of CRC [28–31]. Up to now, surgery, conventional cytotoxic chemotherapy and targeted therapies remain the principal treatment for CRC patients who are at high risk of developing recurrent or metastatic disease [32]. However, the therapeutic effects are still not satisfied, and the metastatic relapse just be reduced by about 7% [33, 34]. The current situation, lack of effective adjuvant therapeutics, demands more understanding of the molecular mechanism involved in the metastatic process for finding new prognostic molecular markers and developing new therapeutic methods.

Emerging research suggests that miRNAs play an essential role in CRC invasion and metastasis [35]. Thus, miRNAs might be proposed as potential diagnostic markers and therapeutic targets for CRC [20]. However, further insights into the roles and molecular mechanisms of miRNAs during CRC progression are needed. The therapeutic utility of miRNAs has been limited given the inefficient delivery of miRNAs into various metastatic tissues. While the liver represents an exception to this rule, because miRNAs tend to accumulate in hepatic tissue [21]. The identification of miRNAs that could suppress liver metastasis would be of great clinical value. We speculate that miR-384 could be utilized as a new miRNA therapeutic target, which might be superior to targeted therapies of single KRAS or CDC42.

Deregulation of miR-384 has only been observed in a few tumor types so far. For instance, a microarray showed that miR-384 was down-regulated in laryngeal carcinoma. Another research implicated mir-384 might play an important role in metastasis of melanoma by binding to the 3'UTR of HDAC3. Recently it was reported that miR-384 exerted tumor-suppressive functions by binding to the 3'UTR of PIWIL4. However, it was unknown whether the dysregulation of miR-384 was associated with the progression of colorectal cancer. Compared with matched adjacent normal tissues, we found that miR-384 was significantly down-regulated in CRC tissues in this study. Moreover, the ectopic overexpression of miR-384 remarkably inhibited the invasion and metastasis of CRC both *in vitro* and *in vivo*. Conversely, the inhibition of miR-384 significantly promoted the invasion and metastasis of CRC cells. Thus, our data demonstrated that miR-384 could suppress the progression of CRC.

Furthermore, we explored the molecular mechanism of miR-384 inhibiting CRC invasion and metastasis. The publicly available bioinformatic algorithms analysis showed that KRAS and CDC42 might be important target genes of miR-384. KRAS oncogene encodes a protein that is a member of the small GTPase superfamily. A single amino acid substitution is responsible for activating mutation. Activation of KRAS promotes the growth and progression in various malignancies, including lung adenocarcinoma, mucinous adenoma, ductal carcinoma of the pancreas and CRC [36]. CDC42 is a member of Rho family of GTPases. And it could be involved in many cellular functions such as cell cycle progression, actin filopodia formation and directional migration [37]. Therefore, both KRAS and CDC42 belong to GTPase superfamily. Although KRAS and CDC42 are known to function in the same signaling cascade, an in-depth investigation into the regulation should be performed. Our study was to identify the miRNA which targets both of these key signaling proteins. It was reported that KRAS gene was mutated in nearly 50% of CRC and played an essential role in the tumorigenesis and progression of CRC resulting from the constitutive activation of Ras signaling [38]. However, as yet specific therapeutic agents against KRAS-mutated CRC have not been developed. Mir-384, targeting the 3'-UTR of KRAS, would be a much better therapeutic target against not only KRAS-mutated CRC but the wild-type KRAS CRC. The change of the KRAS protein, which was the prime therapeutic target for diverse human cancers, will induce the alteration of many key molecules of downstream signaling pathways including Ras-Ral-CDC42. And CDC42 represents a class of Ras-related signaling molecules often deregulated in many human cancers such as testicular cancer, colorectal cancer, breast cancer, head and neck carcinoma and melanoma [39–41]. The up-regulated CDC42 activity may induce a variety of cellular responses including cellular transformation, cell division, cell invasion, migration, invadopodia formation, enzyme activity, filopodia formation, and cell polarity in cells [42]. In addition, deregulation of CDC42 alters the normal functioning of the cells, accounts for the initiation of signaling pathways and correlates with several pathogenic processes of cancer [43, 44]. Consequently, miR-384, targeting both KRAS and CDC42, would play important roles in suppressing the progression of CRC.

In brief, our findings confirmed that miR-384 was down-regulated in CRC. Meanwhile, we have experimentally validated the role of miR-384 in suppressing liver metastasis of CRC cells by targeting KRAS and CDC42. It suggests that miR-384 might be a potent therapeutic target for CRC, and restoration of miR-384 might become a useful therapeutic approach for targeting malignant CRC.

## MATERIALS AND METHODS

### Tissue specimens and cell culture

A total of 100 cases of fresh CRC tissues and their matched adjacent normal tissues were collected after curative surgical operations from the Department of General Surgery, Nanfang Hospital (Guangzhou, China) from February 2014 to January 2015. All tissue biopsies were freshly frozen in liquid nitrogen until further use. None of the patients underwent chemotherapy, radiotherapy and immunotherapy and they had been diagnosed with colorectal columnar adenocarcinoma by Hematoxylin-Eosin (HE) staining. The clinic pathological information including age, gender, differentiation degree and TNM (T, invasive depth; N, lymph node metastasis and M, distant metastasis) stage of the patients were collected. Informed consent was obtained from all patients before surgery informed consent and approval by the ethics committee of Southern Medical University Institutional Board (Guangzhou, China).

The human CRC cell lines SW480, SW620, SW837, HCT116, HCT15, LOVO and normal colorectal mucosa cell FHC were obtained from American Type Culture Collection (ATCC). SW620 and SW837 were cultured in DMEM medium (Invitrogen, Carlsbad, CA, USA) with 10% fetal bovine serum (FBS, Gibco); FHC, SW480, HCT116, HCT15 and LOVO were maintained in RPMI-1640 medium (Invitrogen) with 10% FBS.

### RNA extraction and real-time PCR

Total RNA was extracted from cells and tissues with Trizol reagent (Invitrogen) according to the manufacturer's instruction. Then cDNA was synthesized from 2μg of total RNA and the quantification of miR-384 was performed by the All-in-One TM miRNA real-time PCR Detection Kit (GeneCopoeia, Guangzhou, Guangdong, China). Real-time PCR was performed via the Applied Biosystems 7500 Sequence Detection system, using iQ^TM^ SYBR Green Supermix (BioRad Laboratories, Hercules, CA, USA) containing 5ng cDNA and 10pM of each primer. The cycling conditions were: one cycle at 94°C for 5min; 40 cycles of 95°C for 30s; 56°C for 30s. Melting curve analysis was conducted for each PCR reaction to confirm the specificity of amplification. The concentration of miR-384 was calculated based on the threshold cycle (CT), and the relative expression levels were calculated as 2^–ΔΔCT^ (ΔΔCT= (CT_miR-384_—CT_U6_)_T_—(CT _miR-384_—CT_U6_)_N_) or 2^–ΔCT^(ΔCT =CT_miR-384_—CT_U6_) after normalization with reference to the quantification of U6 small nuclear RNA expression. As for the target genes, RT was conducted with the SuperScript First-Strand Synthesis System for RT-PCR (Invitrogen) according to the manufacturer's protocol. Real-time RT-PCR was conducted by SYBR Green I (Applied BioSystems). The data were normalized to the geometric mean of housekeeping gene GAPDH and calculated as 2^–ΔΔCT.^ The primers were shown in the Supplemental Table S2 (Supplementary Table S2).

### Plasmid construction and transfection

The miR-384 binding site in the KRAS is located at 1589-1594 bp, whose full length of 3'UTR is 4686bp, and at 1329-1334 bp in the CDC42 3'UTR, whose full length is 1440bp. The region of human KRAS 3'UTR at 1474-1917 bp and CDC42 3'UTR at 1106-1524 bp were PCR-amplified and inverted into the Xho I/Not I sites of the psiCHECK-2 luciferase reporter plasmid (Promega). The primers used to construct the plasmid were listed in supplemental material 3(Supplementary Table S3). The KRAS and CDC42 constructs were generated by cloning PCR-amplified full-length human KRAS (G12V) and CDC42 cDNA into Psin-EF-2. MiR-384 mimics, inhibitor and their control oligos, the lentiviral vectors and their paired control lentiviral vector were purchased from Genecopoeia(Guangzhou, China). The Sequences were shown in supplemental Table S4 (Supplementary Table S4). All constructed plasmids were transfected into 293FT cells to produce the lentiviral particles. Then the cells were infected with the retroviral production or purchased relevant miR-384 lentiviral vectors. Retrovirus-infected cells were selected by Puro or Hygro until all of the relative uninfected cells were dead. Real-time PCR and Western blot analysis were performed to confirm the stable expression.

### Western blot

Protein lysates were prepared, subjected to SDS-PAGE, transferred to PVDF membranes, and then blotted according to standard methods with anti-KRAS (Proteintech, USA) and anti-CDC42 (Bioworld Technology, St. Louis Park, MN, USA). Chemiluminescent signals were detected by Supersignal West Pico and exposure to autoradiography (HyBlot CL). A monoclonal anti-a-Tubulin antibody (Sigma, St. Louis, MO, USA) was taken as inner control to confirm the equal loading of protein.

### Wound-healing assay, Transwell migration assay, Matrigel invasion assay and three-dimensional morphogenesis assay

#### Wound-healing assay

Cells were seeded in six well plates and incubated under permissive conditions until 90% confluence. After serum starvation for 24 h, wounds were created in the confluent cells using a pipette tip. Wound healing within the scrape line was then observed and photographed at indicated time points. Each experiment was repeated at least three times.

#### Transwell migration assay

A Boyden chamber with 8μm-pore filter membrane was used. Briefly, cells (1×10^5^) in culture medium containing free FBS were seeded in the upper chamber, and the culture medium with 20% FBS was added in the lower chamber as a chemoattractant. After incubation for 48 h, the chamber was fixed in 4% paraformaldehyde and stained with Haematoxylin. Cells on the upper side of the filter were removed with cotton swabs. Cells that migrated to the lower side were fixed in 4% paraformaldehyde and stained with Haematoxylin. The migratory cells on the lower surface of the filter were counted (10 random 200×fields per well). Three independent experiments were performed and the data were presented as the mean ± s.d.

#### Matrigel invasion assay

Matrigel invasion assay was performed similarly to Transwell migration assay, excepting that the upper side of the filter was firstly coated with 0.2% Matrigel (BD Biosciences, San Jose, CA, USA). The experiment was repeated three times.

#### Three-dimensional morphogenesis assay

Cells (1×10^4^) were resuspended in medium supplemented with 5% Matrigel (BD Biosciences). Then the matrigel-cell mixture was seeded on the top of 24-well plates coated with matrigel previously. The plates were incubated at 37° in a humid atmosphere of 5% CO_2_. Three dimensional morphological structures were observed and the microscopic images were captured at 2-day intervals for 2-3 weeks. The filopodia formed by each cell sphere were counted under microscopy.

### Luciferase assays

Cells were seeded in 24-well plates (1×10^5^/well) the day before transfection. The psiCHECK-2-luciferase reporter gene plasmids psiCHECK-2-KRAS-3^'^-UTR, psiCHECK-2-CDC42-3'-UTR, or the control-luciferase plasmid were transfected into the cells by Lipofectamine 2000 Reagent (Invitrogen). Luciferase activities were assayed 48 hours after transfection by the Dual Luciferase Reporter Assay Kit (Promega) following the manufacturer's instructions. The results were presented after normalization with the measured values of firefly luciferase. All experiments were conducted at least three times and the data were presented as mean±standard deviation (mean±s.d.).

### Animal studies

Animal experiments were performed according to the NIH guidelines for using experimental animals. 6 weeks-old Balb/C athymic nude mice (nu/nu) obtained from the Animal Center of Southern Medical University (Guangzhou, China) were injected intrasplenically with 1×10^6^ SW480/Vector, SW480/miR-384, LOVO/NC, LOVO/miR-384-in, SW480/Vector, SW480/miR-384, SW480/miR-384+KRAS and SW480/miR-384+CDC42. The 1×10^6^ cells were diluted in 200μl RPMI 1640 medium without FBS and then were injected slowly under the spleen envelope. Mice were closely observed when showing significant cachexia. Then the survival time was recorded. All the mice were euthanized 50 days after injection. The liver was excised and the numbers of gross metastatic foci metastases were observed. Then the liver was fixed in 4% paraformaldehyde, embedded in paraffin, and 4μm sections were prepared and stained with HE staining.

### Statistical analysis

All statistical analyses were carried out using the SPSS20.0 for Windows. The data were expressed as means ± standard deviations (SD) from at least three independent experiments. The two-tailed paired Student's t-test was conducted for analyzing two groups. The Mann-Whitney U-test and Spearman's correlation analyses were carried out to analyze the relationship between miR-384 expression and the clinic pathological features of CRC. Survival curves were described by the Kaplan-Meier method and compared with the log-rank test. P<0.05 was considered significant. Statistically significant data were indicated by asterisks: ^*^ (*P* < 0.05), ^**^ (*P* <0.01).

## SUPPLEMENTARY MATERIALS FIGURES AND TABLES


