# Supplementary material for: MiR-384 inhibits human colorectal cancer metastasis by targeting KRAS and CDC42
Source: Oncotarget. 2016 Oct 17;7(51):84826–38. doi: 10.18632/oncotarget.12704 (PMC5356701; doi:10.18632/oncotarget.12704)
Supplement: Supplementary file 1 [file oncotarget-07-84826-s001.pdf]

MiR-384 inhibits human colorectal cancer metastasis by targeting KRAS and CDC42

SUPPLEMENTARY FIGURES AND TABLES

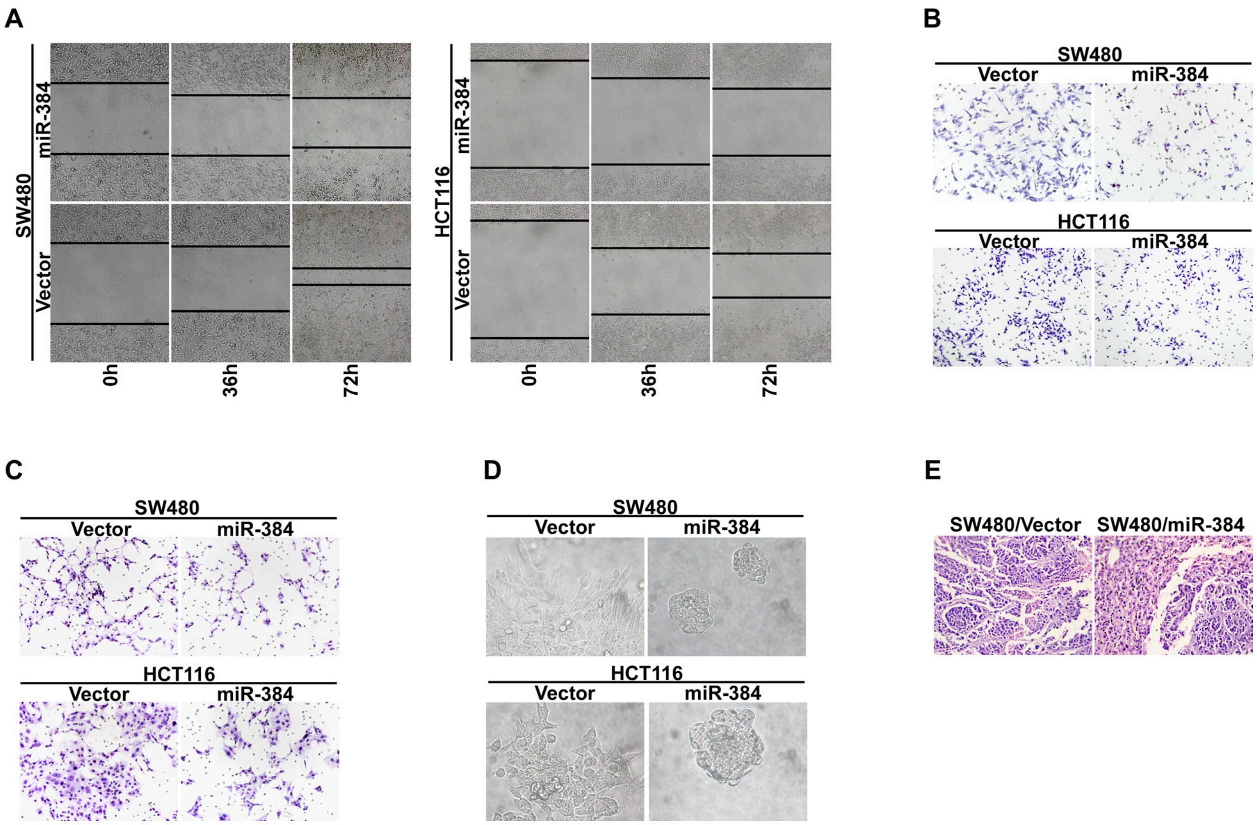

Supplementary Figure S1: A. Representative images of wound-healing assay. Original magnification, ×100. **B-C.** The migratory and invasive properties of SW480/Vector, SW480/miR-384 and HCT116/Vector, HCT116/miR-384 cells were analyzed using Boyden chambers or Matrigel-coated Boyden chambers. Original magnification, ×200. **D.** Representative images of three-dimensional morphologies assay. Original magnification, ×400. **E.** Representative images of HE staining of liver metastatic lesions formed in mice injected intrasplenically with SW480/Vector and SW480/miR-384. Original magnification, ×400.

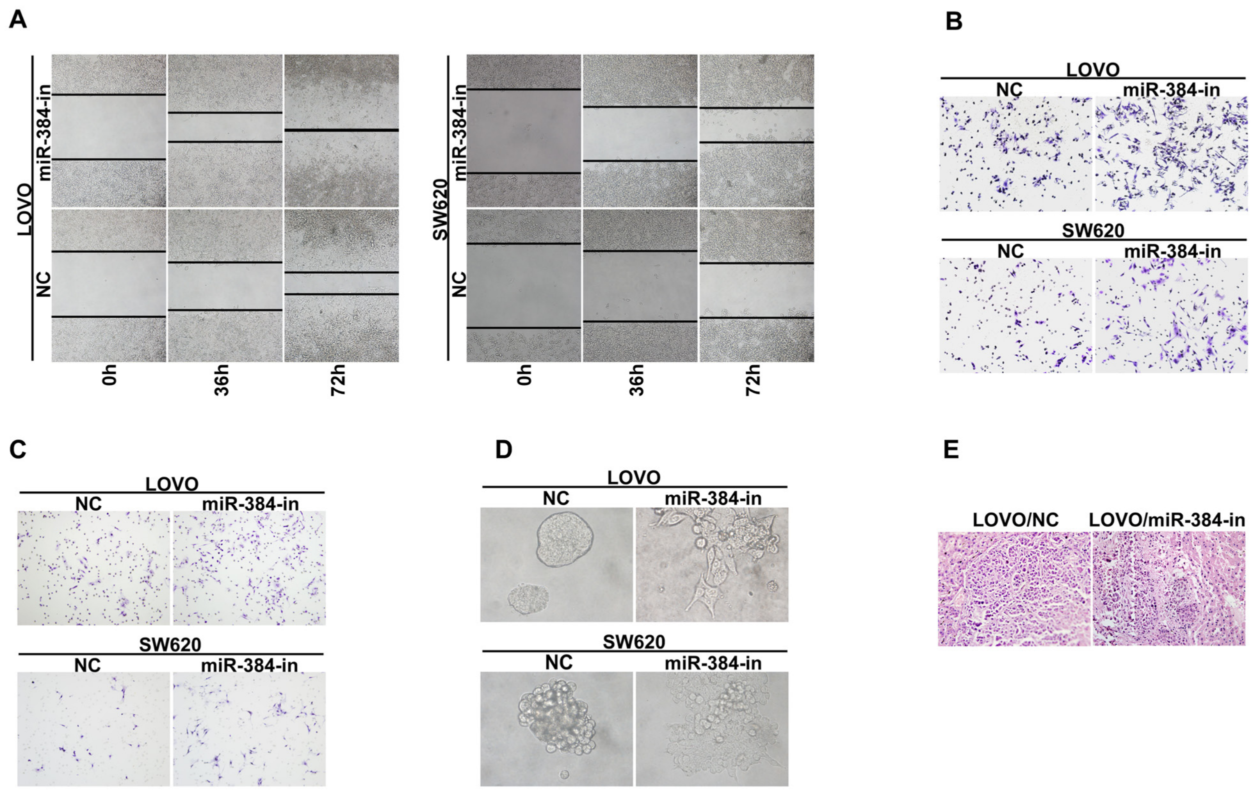

Supplementary Figure S2: A. Representative images of wound-healing assay. Original magnification,×100. **B-C.** The migratory and invasive properties of LOVO/NC, LOVO/miR-384-in and SW620/NC, SW620/miR-384-in cells were analyzed using Boyden chambers or Matrigel-coated Boyden chambers. Original magnification,×200. **D.** Representative images of three-dimensional morphologies assay. Original magnification, ×400. **E.** Representative images of HE staining of liver metastatic lesions formed in mice injected intrasplenically with LOVO/NC and LOVO/miR-384-in. Original magnification, ×400.

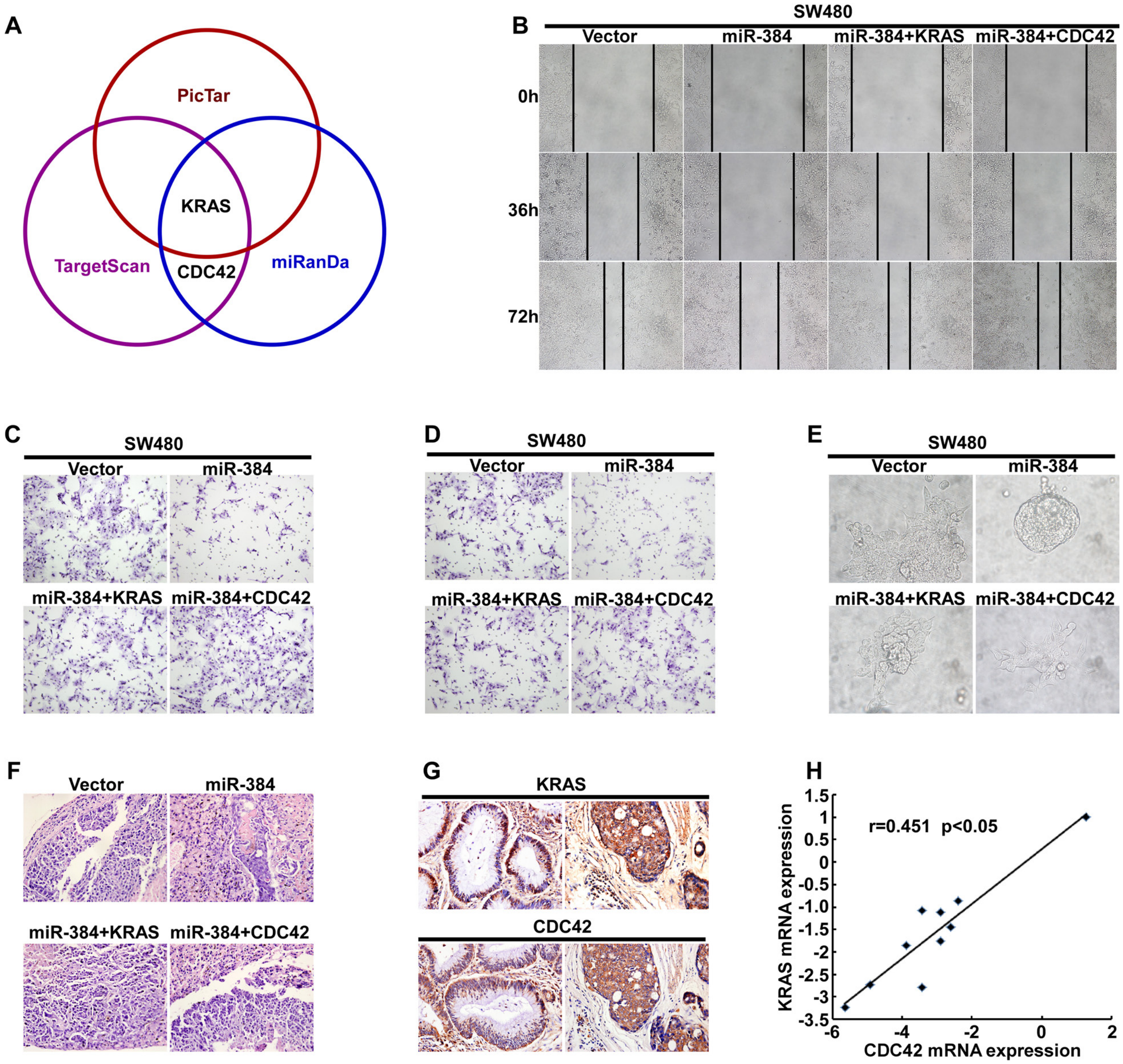

Supplementary Figure S3: KRAS and CDC42 are targets of miR-384 and repression of KRAS and CDC42 plays an important role in miR-384-inhibited invasion and metastasis of CRC cells. A. KRAS and CDC42 are targets of miR-384 according to the publicly available bioinformatic algorithms. **B.** Representative images of wound-healing assay. Original magnification, ×100. **C-D.** The migratory and invasive properties of SW480/Vector, SW480/miR-384, SW480/miR-384+KRAS and SW480/miR-384+CDC42 cells were analyzed using Boyden chambers or Matrigel-coated Boyden chambers. Original magnification,×200. **E.** Representative images of three-dimensional morphologies assay. Original magnification, ×400. **F.** Representative images of HE staining of liver metastatic lesions formed in mice injected intrasplenically with SW480/Vector, SW480/miR-384, SW480/miR-384+KRAS and SW480/miR-384+CDC42. Original magnification, ×400. **G.** The protein expression of KRAS and CDC42 by IHC in CRC tissues (Left, high miR-384 expression; Right, low miR-384 expression). Original magnification, ×400. **H.** Spearman correlation analyses of KRAS expression and CDC42 mRNA expression.

Supplementary Table S1: Spearman correlation between miR-384 expression and Clinicopathologic Characteristics of CRC

Clinicopathological Variables	miR-384 Expression	
	Spearman correlation coefficient	P value
Age	0.088	0.382
Gender	0.084	0.404
Differentiation	0.801	0.000
T classification	1.000	0.000
N classification	0.391	0.000
M classification	0.444	0.000

Supplementary Table S2: Primer sequences used for target gene real-time PCR

Gene	Forward primer	Reverse primer
**KRAS**	GAGTACAGTGCAATGAGGGAC	CCTGAGCCTGTTTTGTGTCTAC
**CDC42**	GAAGGCTGTCAAGTATGTGG	CTCTTCTTCGGTTCTGGAGG
**GAPDH**	GACTCATGACCACAGTCCATGC	AGAGGCAGGGATGATGTTCTG

Supplementary Table S3: Primer sequences used for amplification and plasmid construction

Gene	Forward primer	Reverse primer
**KRAS-3'UTR-WT**	CCGCTCGAG AGGCAGACCCAGTATGAA	GGGGCGGCCGCGACCACCACAGAGTGAGATT
**CDC42-3'UTR-WT**	CCGCTCGAGTCCCTGGTTCCACTCT	AAATATGCGGCCGCATCAGCAAATGGCAAA

Supplementary Table S4: Sequences of miR-384 mimic and inhibitor

Geneb	Sequence	
**miR-384 mimic**	AUUCCUAGAAAUUGUUCAUA	UGAACAAUUUCUAGGAAUUU
**miR-384 inhibitor**	UAUGAACAAUUUCUAGGAAU	
**miR-384 mimic NC**	UUCUCCGAACGUGUCACGU	
**miR-384 inhibitor NC**	CAGUACUUUUGUGUAGUACAA	
